# Developing a good practice model to evaluate the effectiveness of comprehensive primary health care in local communities

**DOI:** 10.1186/1471-2296-15-99

**Published:** 2014-05-15

**Authors:** Angela Lawless, Toby Freeman, Michael Bentley, Fran Baum, Gwyn Jolley

**Affiliations:** 1 Southgate Institute for Health, Society and Equity, Flinders University, GPO Box 2100, Adelaide SA 5001, Australia

## Abstract

**Background:**

This paper describes the development of a model of Comprehensive Primary Health Care (CPHC) applicable to the Australian context. CPHC holds promise as an effective model of health system organization able to improve population health and increase health equity. However, there is little literature that describes and evaluates CPHC as a whole, with most evaluation focusing on specific programs. The lack of a consensus on what constitutes CPHC, and the complex and context-sensitive nature of CPHC are all barriers to evaluation.

**Methods:**

The research was undertaken in partnership with six Australian primary health care services: four state government funded and managed services, one sexual health non-government organization, and one Aboriginal community controlled health service. A draft model was crafted combining program logic and theory-based approaches, drawing on relevant literature, 68 interviews with primary health care service staff, and researcher experience. The model was then refined through an iterative process involving two to three workshops at each of the six participating primary health care services, engaging health service staff, regional health executives and central health department staff.

**Results:**

The resultant Southgate Model of CPHC in Australia model articulates the theory of change of how and why CPHC service components and activities, based on the theory, evidence and values which underpin a CPHC approach, are likely to lead to individual and population health outcomes and increased health equity. The model captures the importance of context, the mechanisms of CPHC, and the space for action services have to work within. The process of development engendered and supported collaborative relationships between researchers and stakeholders and the product provided a description of CPHC as a whole and a framework for evaluation. The model was endorsed at a research symposium involving investigators, service staff, and key stakeholders.

**Conclusions:**

The development of a theory-based program logic model provided a framework for evaluation that allows the tracking of progress towards desired outcomes and exploration of the particular aspects of context and mechanisms that produce outcomes. This is important because there are no existing models which enable the evaluation of CPHC services in their entirety.

## Background

Comprehensive primary health care (PHC) is a model of health system organisation that has considerable promise in addressing 21st century health issues including effective management and prevention of chronic diseases, achieving more equitable health outcomes and involving communities in planning and managing services [1]. Its focus on equity means it is especially suited to developing population health approaches that include special consideration of the needs of population groups who have poorer health status.

We use the term *Comprehensive* PHC (CPHC) to differentiate from PHC which takes a selective approach focusing on interventions targeted at specific diseases or simply describing first line medical care [2-4].

CPHC was the major plank of the Health for All by 2000 strategy that was articulated by the World Health Organization in the Alma Ata Declaration on Primary Health Care in 1978 [5] (for an introduction to the Alma Ata, see [6]). Some of the key elements of the Health for All strategy were:

• CPHC as the backbone of a nation's health strategy with an emphasis on strategies to promote health and prevent disease;

• Recognition that CPHC should be adapted to the particular circumstances of a country and communities within it;

• Achievement of equity in health status; and

• Participation in the planning, organisation, operation, and control of CPHC, supported by appropriate education ([7], p. 515).

The history of CPHC approaches in Australia pre-dates the Alma Ata Declaration through “particularly progressive community health movements, which promoted health centres that attempted to put comprehensive PHC in to practice albeit against the tide of the mainstream health system” ([6,] p. 38). Examples of services operating in the CPHC tradition include multidisciplinary community health centres in Australia which were distinguished from the dominant fee-for-service practices by their focus on local populations, their involvement of local people in their management and programs, the comprehensiveness of the strategies employed including treatment, disease prevention, health promotion and community development and a focus on equity [8,9]. Many facilitated community groups ranging from exercise groups to food cooperatives to parenting support to coalitions against domestic violence to environmental action to name just a few. Likewise Canadian Community Health Centres employ strategies across the continuum from services to individuals to community wellbeing and healthy public policy. Again community governance, teamwork and intersectoral collaboration are features of these centres with attention to provision of services to those most in need and with difficulty accessing services [10]. In the US community health centres employ multi-disciplinary teams to deliver “affordable, comprehensive, coordinated, patient-centered care” with a focus on community accountability and cultural competence [11]. Services include translation, interpretation, and transport. These centres reflect the trend of CPHC implementation in high income countries where the emphasis is on increasing access to a range of health services and implementing programs addressing the social determinants of health [4].

Although CPHC has remained on the margins of health care systems in most high income countries, the mixed progress in improving health and especially in addressing chronic disease has led to a re-examination of comprehensive primary health care approaches [1,12]. The World Health Organization called for a revitalization of primary health care in the 2008 World Health report [1] and the Commission on the Social Determinants of Health [13] recommended it as the basis for health systems in order to achieve health equity. The shortcomings of selective and disease centred approaches have become apparent with the development of a patchwork of health interventions lacking coordination and sustainability [14,15]. There is a considerable literature regarding implementation and outcomes of individual components of PHC (e.g., action to improve equity of access) but a global literature review of CPHC found little literature that evaluates CPHC as a service model [4,16]. The review did document the accumulating evidence of positive impacts on community and intersectoral processes and cost effectiveness. These effects increase as the degree of comprehensiveness of PHC increases [4]. Evaluation approaches that have focused on individual components of models independently, rather than examining their internal coherence and incorporating the effects of context, contribute to the lack of consensus regarding exactly what constitutes PHC [17].

The research reported in this paper is part of a five year project funded by the National Health and Medical Research Council of Australia that sought to contribute to the understanding of CPHC by studying models of CPHC services in the Australian context and trialing evaluation methods to determine the effectiveness of CPHC services. This paper focuses on the first stage of the project, in which we developed a model of CPHC (combining program logic and theory-based approaches) applicable to the Australian context. We were seeking to answer three inter-related questions:

1. What are the characteristics of Australian CPHC that need to be captured in a model?

2. What processes are required to develop such a model?

3. Does use of a theory driven program logic model provide a means of describing CPHC as a whole that can be used as a basis of evaluation?

## Methods

This work was undertaken as part of a five year program of work studying models of CPHC services in the Australian context and developing evaluation methods to determine the effectiveness of CPHC services. The study examines six Australian case study sites providing differing models of CPHC, some inclusive of primary medical care, with a mix of funding and management models (see Table 1). The sites ranged from longstanding examples of CPHC to newly emerging models, and included an Aboriginal community controlled service (Congress), a sexual health non-government organisation (SHineSA), and four services directly managed by the South Australian government. The South Australian state government funded and managed services are anonymised as Services A, B, C, and D. The researchers have long-standing relationships with the study sites that facilitated our engagement with them.

**Table 1:** Characteristics of the six case study sites, 2010

	**Approximate # of staff (FTE)**	**Budget (p.a.)**	**Main source of funding**	**Examples of disciplines employed**
Service A	16 (13.5)	$1.2 m	SA Health	Social worker, nurse, speech pathologist, occupational therapist, dietitian, cultural worker, lifestyle advisor
Service B	26 (20)	$1.1 m	SA Health	Medical officer, lifestyle advisor, PHC worker, podiatrist, nurse, speech pathologist
Service C	36 (22)	$1.7 m	SA Health	Nurse, dietitian, speech pathologist, psychologist, occupational therapist, cultural worker, social worker
Service D	12 (10.8)	$0.5 m	SA Health	Aboriginal health worker, PHC worker
Congress	320 (188)	$20 m	Dept. of Health & Ageing	Medical officer, psychologist, social worker, youth worker, midwife, nurse, Aboriginal health worker, pharmacist
Shine SA	100 (55)	$6.1 m	SA Health + Dept. of Health & Ageing	Medical officer, nurse, counsellor, workforce educator, community health worker, disability worker, Aboriginal educator, multicultural worker

Weiss [18] suggests that when developing a theory-based evaluation the underpinning theory can be drawn from the literature, or where programs are the products of history, experience, and intuition, the theory can be drawn from those associated with the program such as the staff and policy makers.

Members of our research team were principal investigators and researchers involved in a global systematic review of published and ‘grey’ literature documenting, evaluating or describing attempts to implement CPHC [4]. A critical literature review and synthesis of description and effectiveness of CPHC in Australia had been conducted in 2007 [16] and the team was able to draw on this as well as undertaking an ongoing scan of the Australian CPHC literature and relevant national and state policies.

At each of our study sites, 7–15 semi-structured interviews (depending on the size of the service) were conducted, totalling 68 in all. The mix of primary health care workers interviewed reflected the spread of disciplines employed across the sites and included dietitians, occupational therapists, speech pathologists, psychologists, social workers, Aboriginal Health Workers, medical officers, lifestyle advisors, nurses, and counsellors. In addition, six regional health executives and two central health department bureaucrats with responsibility for primary health care services were interviewed.

The interview data, the CPHC literature and the extensive experience of the research team in working in, and with, primary health care services was used to develop a rough draft model using widely employed program logic model conventions (see for example [19]) to provide a visual depiction of a CPHC service. This model was devised as a starting point for discussion and further development of the CPHC model with the services and the research reference group.

A series of workshops to inform the development of the model were planned with our study sites. A workshop process was developed and piloted with a primary health care service that was not one of our six study sites and a small cross-agency forum. The process was found to encourage robust debate and provided useful feedback regarding modification to the model. A workshop was then held at each of the participating sites and attended by managers, practitioners and administration staff. The sites have vastly different staffing numbers (ranging from approximately 320 at the largest site to 12 at the smallest). At smaller sites, a high percentage of the total staff were able to attend; for larger sites we aimed to ensure that key positions and interests were represented, that is, participants were drawn from different teams and professions.

The workshops elicited much discussion and debate on the draft model. Including a range of professions and practitioners who used a range of strategies – from individual treatment to community development activities – in the workshops proved a useful way of ensuring the model captured the ‘complex and messy’ ([20], p. 721) nature of CPHC. Much discussion centred on the exact language to be used to describe the principles and activities of CPHC. In particular participants were keen for the model to reflect a social rather than a biomedical view of health. Thus the inclusion of language in the draft model that was seen to derive from a medical rather than social view of health was vigorously challenged. For example, the first draft of the CPHC model used the word treatment rather than care and this was seen as inappropriate when applied to areas such as domestic violence interventions.

We were particularly tested in trying to portray the interaction of model components graphically whilst maintaining the model’s readability and utility as an evaluation framework. Various means of capturing this were trialled and then discarded or modified (for example, key components were at one stage depicted as interlocking cogs but later discarded as it drew criticisms of appearing too mechanistic).

In between workshops, points raised and any resulting modifications to the model were discussed and debated in research team meetings. In these meetings researchers also incorporated insights from the analysis of interview data. Modifications made to the CPHC model were presented back to staff at a subsequent workshop where the changes were endorsed or further discussed. In three sites, a third workshop was held before the model was approved by participants. In addition, models were circulated by email and feedback invited. We provided feedback to participants on the issues raised at other sites, which encouraged shared learning between services. This consensus-building approach to the model development built a high level of engagement with managers and practitioners at the study services.

A generic model was developed to explain the overall operation of CPHC and site–specific models with detailed local program information were prepared for each site. The models were endorsed at a research symposium involving all investigators, members of the study’s Critical Reference Group (which was established to provide input from a range of experts in primary health care policy and service provision), and key stakeholders. This paper presents the generic model which can be adapted for CPHC services to reflect their particular context and mode of operation.

Ethics approval was received from Flinders University’ Social and Behavioural Research Ethics Committee and the Aboriginal Health Research Ethics Committee (South Australia).

## Results

**Figure 1:**
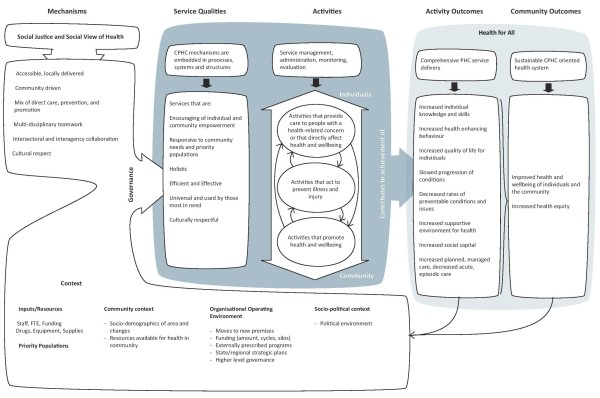
The Southgate model of comprehensive primary health care in Australia.

The model of CPHC for the Australian context developed through our process of consultation is presented in Figure 1. Drawing on a realist approach, the model “explores the relationship … among “context” (the study’s organizational setting and external constraints, including financial and human resources, prevailing policies, and technologies), “mechanisms” (the stakeholders’ ideas about how change will be achieved in an intervention), and “outcomes” (the intended and unintended consequences of the change efforts)” ([21], p. 396). CPHC mechanisms and context are theorised as interacting to create spaces for action which may enable or constrain the service qualities that should characterise a CPHC service and which, in time, contribute to individual and community health and equity outcomes.

### Mechanisms

In attempting to capture the mechanisms of CPHC, our starting point was that CPHC is based on a philosophical framework incorporating key principles that underpin service development. CPHC is based on the assumption that outcomes such as empowerment of individuals and communities occur *because* of the principles being embedded in practice. For example, any service providing primary care will deliver individual treatment sessions, but if the service claims to use a CPHC approach then the incorporation of CPHC principles should be evident. We could ask, for instance, ‘how does CPHC’s concern with equity figure in individual treatment?’ For this example, it might be through the presence of a priority of access scheme, or outreach sessions for particular population groups [22].

The outcomes of the service are not simply related to events or behaviours but are the result of ‘complex transactions of many different kinds of structures at many different levels’ ([23], p. 805). CPHC principles, when operationalised, trigger generative mechanisms - ‘underlying entities, processes, or structures which operate in particular contexts to generate outcomes of interest’ ([3], p. 368). For example, it is not always clear why an apparently accessible health service is not used by some groups in society despite their evident need. We may theorise that this is because marginalised groups are unlikely to articulate their needs but paradoxically are unlikely to participate in programs unless they are actively involved in their design and implementation [24]. If strategies (such as cultural respect strategies, making the health centres welcoming of diversity, activities such as community lunches or playgroups to engage and meet members of the community, or having board of management representatives from the community) are put into place to address barriers that act to exclude particular groups - operationalising a PHC principle - it may act to trigger a shift in power relations. The result may be individual and community empowerment and an increased likelihood that the group will participate in services and programs.

A high level of agreement on the principles of CPHC as presented in the draft CPHC model was gained. This consensus may well be an artefact of the history and structure of primary health care in Australia and the particular histories of our study sites. Notions of equity, community participation, multidisciplinary teamwork and intersectoral collaboration featured in the early development of the Aboriginal community controlled and South Australian community health sectors, and have continued to shape ideas about CPHC. Practitioners who choose to work in community controlled or state funded sites rather than fee for service private practice may well be those who are committed to CPHC values in the Alma Ata tradition.

The agreed operating principles are that CPHC services should be: accessible, locally delivered; community driven; comprise a mix of direct care, prevention, and promotion; characterised by multi-disciplinary teamwork and intersectoral and interagency collaboration; and are culturally respectful. These principles are informed by a concern with social justice and a social view of health.

### Context

In the tradition of qualitative research, context is understood as integral to understanding the phenomenon being studied and central to the analysis and interpretation of results [25]. As Pawson and Tilley [26] observe, programs operate according to the conditions into which they are placed. Context does not operate as a passive backdrop to action but dynamically affects causation, as favourable or unfavourable conditions may determine whether or not a particular mechanism is triggered.

In Australia the health system is shaped by the division of powers whereby responsibility for the system is split between the Commonwealth Government and State and Territorial Governments. In 2007, a new Australian federal Labor Government was elected with a mandate for health reform. In 2010, the Commonwealth Government launched their plans for PHC reform describing them as ‘Australia’s First National Primary Health Care Strategy’ [27]. A national network of PHC organisations termed ‘Medicare Locals’ was phased in with the first tranche of Medicare Locals beginning operation in mid 2011. Changing health policy direction has resulted in shifting priorities, and a realignment of state and Commonwealth arrangements. In South Australia at least, the Commonwealth funded Medicare Locals are now seen to be the health agency with major responsibility for health promotion activity resulting in the state-funded services withdrawing from that space.

At the state level, a number of changes and new initiatives have an impact on PHC including the 2007 *GP Plus Health Care Strategy*[28] which has seen an increased emphasis on community and home care, aiming to keep people out of hospital and reduce hospital stays. Associated with this strategy new centres have been built resulting in relocation and reconfiguration of some services. Three of our research sites have relocated and a fourth in effect merged with another. The South Australian health system has been subject to repeated reorganization with changes to health regions and governance structures. There has been considerable turnover of staff at managerial and regional levels. Individual service boards of management were abolished in favour of regional boards which themselves were later dismantled. The *SA Health Care Plan 2007–16*[29] focuses on lifestyles, information campaigns, and behavioural health promotion. Services are required to deliver more centrally prescribed programs aiming, for example, at combating childhood obesity or addressing chronic conditions.

The site located in the Northern Territory has also been affected by waves of reform. Whilst recent changes have increased funding to Aboriginal health, much of it has been tied to prescribed programs rather than locally determined services.

Participants argued strongly that the changing political and bureaucratic imperatives created a dynamic context which shaped who received what services and the way in which services were delivered. The constraining impact of decisions from the central health bureaucracy regarding funding, policies, structures and processes which result from waves of reform was particularly stressed by the state-funded sites. Participants in the workshops often noted that the practice they were actually engaged in differed from the ‘good practice’ they wanted depicted in the model. Recent changes were seen to privilege a risk factor and lifestyle approach to prevention and health promotion and, at least in the eyes of many participants, services had retreated from the empowerment agenda of earlier PHC efforts [30].

### The “space for action”

Context and mechanisms interact to form the ‘space for action’ in which services aspiring to CPHC have the opportunity to foster service qualities consistent with CPHC principles. In this space for action, it is the dynamic relationship between mechanisms and context that enables or constrains the extent to which mechanisms lead to success or disappointment and in which desirable service qualities are manifested. Our study sites provide examples of differences in this space for action. For example, Congress staff have been involved in addressing policy issues related to the supply of alcohol in their communities. Community governance provides a supportive context for staff to look beyond individual alcohol issues and work in collaboration with a range of other stakeholders to advocate for change to the systems and structures that contribute to people’s behaviour. An example of this is Congress’s membership of The People’s Alcohol Action Coalition which has successfully advocated for constructive reforms to the sale of alcohol such as the implementation of a voluntary ‘floor price’ reducing the availability of cheap alcohol in Central Australia. Their ‘space for action’ supports such initiatives. In contrast, A B C and D site staff reported the operating climate constrained their ability to address broader social health issues. Their space for action is considerably cramped in comparison to Congress.

State managed services also noted that they were sometimes required to deliver programs or services in a way they felt ignored local conditions. A program aimed at reducing childhood obesity through parental engagement was required to be delivered in a standard format even when those delivering the program judged it not to be appropriate (e.g., using written materials with low-literacy participants). Drawing on Greenhalgh et al’s [21] realist analysis of constraining or enabling factors interacting with mechanisms to lead to either disappointment or success, The childhood obesity example demonstratesthat the mechanism “community driven” was highly constrained by the imposition of a standard format program..

Where CPHC mechanisms operate in a supportive context, we postulate that they produce particular service qualities: encouraging of individual and community empowerment; responsive to community needs and priority populations; holistic; efficient and effective; used by those most in need and culturally respectful.

### Activities

In keeping with the Alma Ata vision [5] of CPHC, and its re-endorsement by the World Health Organization in 2008 [1], the activities depicted in the model span a continuum from individual treatment and rehabilitation to the salutogenic notion of promoting good health. Three overarching strategies are identified: activities that provide care to people with a health-related concern or that directly affect health and wellbeing; activities that act to prevent illness and injury; and activities that promote health and wellbeing. Action across the continuum is important if population health is to be improved and health inequities overcome [31]. Participants strongly endorsed the need for activities across the continuum to be included in a model of CPHC even though the current operating context shifted practice to the treatment end of the continuum and constrained health promotion efforts.

### Outcomes

A CPHC service characterised by the service qualities listed above is theorised to contribute to improving the health and wellbeing of the community and increasing health equity. Using the logically causal pathway progress toward these ‘big picture’ outcomes can be tracked through achievement of more proximal outcomes such as increased individual knowledge and skills; increased health enhancing behaviour; improved quality of life for individuals; slowed progression of conditions; decreased rates of preventable conditions and issues; increased supportive environments for health; increased social capital; increased planned, managed care; and decreased acute, episodic care.

## Discussion

In developing a model of CPHC for the Australian context, we were endeavouring not simply to describe activities, products or outputs but to also provide an explanation of how CPHC operates to bring about desired ends such as improved individual and population health and increased health equity. Logic models can elaborate program theory [32] revealing how programs are thought to work through an examination of their underlying mechanisms. Although program logic and program theory are sometimes used interchangeably, Leeuw [33] draws a distinction between the two, noting that program logic specifies linkages between inputs, components and outcomes but rarely details the underlying mechanisms assumed to be responsible for those linkages.

The model also depicts the dynamic relationship between particular conditions or circumstances and the mechanisms. “(I)nnovations, programs, and interventions will work only in particular circumstances and … the purpose of the evaluation is to find those conditions: Which mechanisms work, in which contexts, and to produce which outcomes?” ([21], p. 396).

The construction of ‘plausible and defensible’ ([34], p. 285) models of the theory underlying the CPHC services (i) allows us to understand and describe how CPHC is thought to work, (ii) enables prioritisation of research questions, choice of data collection and analysis methods [3] and (iii) helps determine the focus of research [26].

We have used the model as the basis for evaluation in two ways: firstly by seeking empirical evidence of change along the causal pathway described by the model; and secondly by providing a framework to examine the relationships between the components, asking how their interaction is likely to lead to favourable or disappointing outcomes.

The achievement of short and long-term outcomes is predicated on the achievement of other links in the chain. This is well summarised by Dwyer, Silburn et al. [35]:

‘…desired outcomes such as improved health status and wellbeing are premised on the generation of certain impacts, such as changes in modifiable risk and protective factors operating in individuals and environments. These impacts are premised on changes in processes and/or structures such as improved capacity and higher quality or better coordination of services and programs. In turn, the implementation of new processes and structures requires a range of inputs or activities such as supporting policy directions, workforce development and funding. These chains of inputs and effects take place in a wider social and political context that mediates the effectiveness of all elements. If empirical evidence of change can be seen for each of the points along the continuum, then it can be reasonably predicted that the outcomes are at least in part attributable to the program’ (p. 12).

A range of indicators that act to identify change along the continuum have been identified and these are being used to assess the extent to which the case study sites conform to or differ from the model of CPHC in the scope and nature of their treatment, rehabilitation, disease prevention and health promotion activities. Provision of access to coordinated comprehensive primary health care services that also addresses population health remains difficult even in countries with advanced health systems [36]. The implementation of CPHC faces many challenges, including the globalisation of market-driven health systems and the privileging of selective approaches to primary health care [4]. Globally, most implementation of PHC has, in fact, been along a continuum from primary medical care to selective PHC to partial implementation of CPHC with few examples of full implementation of CPHC.

The model also allows us to examine the relationship between the various components of the model. As noted above, the ‘space for action’ results from the interaction between mechanisms and context. Exploring the relationships between components allows us to ask what factors relating to context and mechanism lead to ‘success’ or ‘disappointment’ in terms of the observed outcomes [21]. By focusing on internal coherence, and accounting for the effect of context, the program logic model provides a robust framework to guide the evaluation of comprehensive primary health care services. As well as production of the model, the process of model development had a number of benefits. Although it was time and resource intensive to gain input from stakeholders in a range of positions (i.e., practitioners, managers, policy makers), the process ensured we had access to appropriate and adequate information to build a model articulating a plausible program theory [37]. The iterative and participatory approach to model development provided a means of testing and retesting ideas and linkages. As Befani and colleagues [38] found, it was necessary to ‘climb up and down the ladder from theory to empirical cases several times’ (p. 190) during this process. Brazil et al. [39], arguing for the importance of theory in health services research, suggest that collaborative research processes such as these are of mutual benefit to researchers and decision-makers alike. They also note that such benefits are derived from strong collaborative relationships that are developed over time rather than as one-off events.

The experience gained in this study reinforces the notion that stakeholder engagement in research, whilst being resource intensive, can also be highly productive [40]. Our long-standing relationships with the services provided the basis for a productive collaboration leading to ‘knowledge with tangible practical consequences’ ([41], p. S2:2).

The constant churning of the health system made engaging with ‘programmes that are entangled in complex, inherently political processes’ ([42], p. 487) difficult. The researchers were well aware they were working in a politicised environment where programs are ‘proposed, defined, debated, enacted, and funded through political processes, and in implementation they remain subject to pressures - both supportive and hostile’ ([43], p. 94). In addition to stakeholder engagement shaping the model, the collaborative relationships forged assisted the researchers to respond to frequent changes in the policy and service context.

### Limitations

As noted previously, our study took place in sites that had implemented CPHC in the Alma Ata tradition over several decades. The high degree of consensus gained in this study may not be replicated in other sites. The structure of the Australian health system meant that medical practitioners were under-represented in the study participants as the majority of medical practice is undertaken in fee-for-service medical practices rather than in state-funded centres.

## Conclusion

The model articulates the theory of change embedded in CPHC services in the Australian context, identifying outcomes, the strategies and activities undertaken and how these strategies and activities were intended to bring about desired changes. The process engaged a wide range of health service staff, regional health executives and central health department staff in the research process and ensured the model reflected current understandings of good practice in CPHC as accurately as possible. The model provides one means for services aspiring to achieve improved community health through the provision of CPHC rooted in the principles of the Alma Ata Declaration to evaluate their progress toward that objective.

## Competing interests

The authors declare that they have no competing interests.

## Authors’ contributions

AL contributed to research design, data collection, analysis, and led the drafting of the manuscript. TF contributed to data collection, analysis, and drafting of the manuscript. MB, FB, and GJ contributed to research design, data collection, analysis, and drafting of the manuscript. All authors read and approved the final manuscript.

## Pre-publication history

The pre-publication history for this paper can be accessed here:

http://www.biomedcentral.com/1471-2296/15/99/prepub
